# The role of metabolic crosstalk between Intestinal epithelial cells and immune cells in inflammatory bowel disease and targeting strategies

**DOI:** 10.3389/fimmu.2026.1854880

**Published:** 2026-09-28

**Authors:** Heng Li, Beibei Wang, Xianhong Shi, Lulu Tian, Bin Li, Tianqi Zhou, Mingcan Qiu, Jiwei Yuan, Kai Yin

**Affiliations:** 1 Department of General Surgery, Affiliated Hospital of Jiangsu University, Jiangsu University, Zhenjiang, Jiangsu, China; 2 Department of General Surgery, Affiliated Hospital of Jiangsu University, Institute of Digestive Diseases, Jiangsu University, Zhenjiang, China

**Keywords:** immune cells, immunometabolism, intestinal epithelial cells, metabolic reprogramming, mitochondrial dysfunction, therapeutic targets

## Abstract

Inflammatory bowel disease (IBD) is a chronic immune-mediated inflammatory disorder characterized by disruption of the intestinal epithelial barrier and dysregulated immune responses. IBD pathogenesis is intricately linked to the metabolic reprogramming of the intestinal epithelial cells (IECs), which are central to both barrier maintenance and immune crosstalk. Mitochondrial dysfunction in the IECs leads to energy deficit, oxidative stress, and barrier failure, which activates the adjacent immune cells and establishes a vicious cycle of inflammation. This review focuses on the key role of the mitochondrial metabolism in IBD with a specific focus on the remodeling of the glucose, lipid, and amino acid metabolic pathways in epithelial and immune cells. We elaborate on the specific regulatory mechanisms underlying these metabolic processes and the functions of metabolic products as signaling molecules in cell–cell communication. In addition to causing energy crises and barrier defects in epithelial cells, metabolic remodeling directly controls the activation of immune cells and their polarization toward a pro-inflammatory phenotype. Thus, a deeper understanding of mitochondrial metabolism underlying IBD would provide a crucial theoretical foundation for developing novel precision therapies targeting metabolism. Additionally, elucidating these metabolic networks would provide new therapeutic opportunities for restoring homeostasis in IBD.

## 1 Introduction

Inflammatory bowel disease (IBD), which includes Crohn’s disease (CD) and ulcerative colitis (UC), is a chronic relapsing inflammatory disorder of the gastrointestinal tract (1). Its etiology involves genetic susceptibility, environmental triggers, and dysregulated immune responses against commensal microbiota (2). Although genome-wide association studies have identified numerous IBD susceptibility genes, many of which are involved in innate immunity, autophagy, and epithelial barrier function, the precise underlying pathogenic mechanisms remain unclear.

Recent immunometabolic analyses have shown that cellular metabolic states profoundly influence immune function and tissue homeostasis (3). Intestinal epithelial cells (IECs)—particularly the stem cells at the crypt base—and the energy-dependent Paneth cells require efficient mitochondrial metabolism to sustain renewal, barrier integrity, and secretory functions including antimicrobial peptide production (4). Similarly, the activation, differentiation, and effector functions of immune cells are closely coupled with their metabolic status. The gut is a metabolically active organ that is constantly exposed to dietary antigens and microbial products. Consequently, it depends on healthy mitochondria for its proper functioning in both the epithelial and immune compartments (3, 5).

Mitochondria are not merely ATP generators. They are signaling hubs that integrate diverse cellular signals and coordinate adaptive responses (6). In the intestinal microenvironment of IBD, inflammatory cytokines impair epithelial mitochondrial function, leading to barrier disruption and increased bacterial translocation. These antigens activate immune cells, which switch to glycolysis and release large quantities of inflammatory cytokines that further exacerbate epithelial metabolic damage (3, 5). Furthermore, abnormal metabolite accumulation and disrupted signaling interfere with the homeostatic crosstalk between epithelial and immune cells. This prevents the timely resolution of inflammation.

Here, we hypothesize that IBD is fundamentally an immune-mediated inflammatory disease driven by a self-amplifying cycle of epithelial mitochondrial dysfunction and aberrant immune activation. This cycle operates through three interconnected phases: (i) initiation, where genetic or environmental insults impair epithelial mitochondrial function; (ii) propagation, where mitochondrial dysfunction disrupts barrier integrity and licenses immune activation; and (iii) amplification, where immune-derived cytokines further damage epithelial mitochondria, which perpetuates the cycle even after the initial trigger subsides. In this model, metabolic reprogramming is not a passive consequence but an active driver that provides energy for cellular functions, directly regulates cell fate through metabolic intermediates, and coordinates intercellular communication (3, 5). Therefore, re-examining IBD pathophysiology from a mitochondrial metabolism perspective may help identify new therapeutic entry points such as the modulation of glucose, lipid, or amino acid pathways or targeting of key metabolic signaling molecules to restore metabolic balance and break the inflammatory cycle (7).

This review examines the mitochondrial metabolic crosstalk between IECs and immune cells in IBD. We discuss mitochondrial dysfunction, metabolic remodeling in epithelial and immune cells, and the therapeutic strategies and challenges arising from these insights.

## 2 Mitochondrial homeostasis and dysfunction

### 2.1 Structural and functional compartmentalization

The mitochondrion comprises an outer and an inner membrane, which create two aqueous compartments, namely, the intermembrane space and the matrix (8). The outer membrane is freely permeable to small molecules, whereas the inner membrane is highly folded into cristae that exhibit a topology characterized by vastly expanded surface area to accommodate the electron transport chain complexes and ATP synthase (9). These cristae are not passive structures; in fact, their shape directly influences oxidative phosphorylation (OXPHOS) efficiency. The matrix houses the TCA cycle enzymes, mitochondrial DNA (mtDNA), ribosomes, and calcium stores, making it the primary site for substrate oxidation and metabolic precursor generation. This spatial segregation of substrate processing in the matrix and electron transfer and ATP generation at the cristae enables an ordered high-efficiency energy conversion that distinguishes mitochondria from other organelles.

### 2.2 Mitochondrial dynamics, quality control and danger signaling

Mitochondria exist as a dynamic interconnected network, and their shape and function are continuously remodeled by opposing fusion and fission events (10). Fusion is mediated by MFN1/2 on the outer membrane and OPA1 on the inner membrane, and it enables content mixing between organelles, which helps dilute localized damage, stabilize mtDNA, and optimize respiratory efficiency. Fission is driven largely by DRP1, and it segments the network, which enables the segregation of dysfunctional subdomains for subsequent clearance. Neither process is merely structural; both directly influence cristae architecture, OXPHOS capacity, and reactive oxygen species (ROS) production.

However, damage exceeding the repair capacity activates quality-control mechanisms. The best-characterized pathway is PINK1/Parkin-mediated mitophagy, where loss of membrane potential stabilizes PINK1 on the outer membrane, which recruits Parkin to ubiquitinate mitochondrial proteins, thereby marking the organelle for autophagosomal engulfment and degradation (11). Furthermore, the mitochondria deploy additional safeguard systems such as the mitochondrial unfolded protein response (UPRmt), which alleviates proteotoxic stress by inducing chaperones and proteases (12, 13). Furthermore, excessive damage triggers the release of mtDNA into the cytosol, which engages the cGAS-STING pathway to drive type I interferon responses (14, 15).

In the intestinal epithelium, the fusion–fission equilibrium is under constant challenge from fluctuating microbial metabolites, hypoxia, and inflammatory mediators. Disruption of this balance—particularly DRP1-driven excessive fission—has been documented in IBD mucosa with correlations to fragmented mitochondrial morphology, impaired bioenergetics, and amplified inflammatory signaling (16). Whether restoring dynamic equilibrium, enhancing mitophagic clearance, or suppressing mtDNA-dependent cGAS-STING activation provides therapeutic benefit remains unknown, but emerging evidence indicates that mitochondrial quality control is a critical node in intestinal homeostasis.

### 2.3 Bioenergetics and redox signaling: ROS as a double-edged sword

In addition to generating ATP, OXPHOS continuously produces reactive oxygen species (ROS) as natural by-products of electron transfer. For decades, this process was viewed simply as inevitable collateral damage, although this perception has now changed. At low physiological concentrations, ROS function as secondary messengers that modulate cell proliferation, differentiation, and adaptation to stress (17, 18). Sies and Jones coined the term “oxidative eustress” to describe this beneficial signaling mode and distinguish it from “oxidative distress,” which is the damaging state that occurs when ROS production overwhelms antioxidant defenses (19). The distinction is not semantic and directly influences the interpretation of mitochondrial dysfunction in IBD.

This duality is especially pronounced in the intestinal epithelium. Moderate ROS generation supports normal crypt homeostasis and wound-healing responses, whereas excessive ROS generated by mitochondrial complex I/III dysfunction, NADPH oxidase 1 (NOX1) upregulation, or glutathione depletion inflicts oxidative damage on lipids, proteins, and mtDNA. Consequently, NOD-like receptor family pyrin domain containing 3 (NLRP3) inflammasome activation is triggered along with the amplification of local inflammation (20, 21). Notably, the relationship between ROS and inflammation is bidirectional. As a result, inflammatory cytokines further impair mitochondrial respiration, which creates a self-reinforcing loop that sustains oxidative stress even after the initial trigger subsides.

The hypoxia-inducible factor (HIF)-1α demonstrates the protective side of this redox circuitry. In the inflamed gut, tissue hypoxia stabilizes HIF-1α, which upregulates barrier-protective genes and promotes epithelial survival (22, 23). In murine models, the conditional deletion of HIF-1α in IECs exacerbated colitis, whereas pharmacological stabilization of HIF-1α via prolyl hydroxylase inhibitors ameliorated the disease (24, 25). These observations challenge the assumption that mitochondrial dysfunction and its redox consequences are uniformly detrimental. The outcome depends on the magnitude, duration, and cellular context of the ROS signal. This contextual dependency complicates therapeutic targeting because the strategies that broadly scavenge ROS may inadvertently suppress the protective signaling. Therefore, interventions with high promise need to selectively curb pathological ROS sources while preserving physiological redox circuits.

### 2.4 The Three faces of mitochondrial dysfunction in IBD

Mitochondria are not passive components of inflammatory signaling but rather actively shape the inflammatory trajectory. In this context, clarity is needed on whether mitochondrial dysfunction initiates IBD or creates a permissive metabolic state that lowers the threshold for inflammation or is simply a consequence of chronic tissue injury. The three possibilities are not mutually exclusive, and the balance among them probably shifts across disease stages and between cell types.

Several study findings support an initiating role. Genetic variants that affect mitochondrial genes including *PARK7* and *ACADM* have been linked to IBD susceptibility, which suggests that primary mitochondrial defects may predispose to intestinal inflammation (26). However, epithelial-specific deletion of the mitochondrial chaperone Hsp60 in mice disrupted OXPHOS and spontaneously triggered microbiota-dependent ileitis, independent of external inflammatory stimuli. This finding directly implicates an initiating role (27). Similarly, loss of STARD7, which is a protein required for maintaining mitochondrial architecture in IECs, increased colitis susceptibility. This reinforces the assertion that intrinsic mitochondrial fragility may act as a disease-initiating lesion (28).

However, mitochondrial dysfunction may additionally act as a permissive state, in which a metabolic vulnerability may amplify responses to otherwise sub-threshold insults. For example, high-fat diets and antibiotic exposure cooperate to impair mitochondrial bioenergetics in the intestinal epithelium, thereby creating a state where subsequent mild inflammatory trigger produces an exaggerated response (29). In this context, mitochondrial dysfunction lowers both the metabolic and physical barriers that normally contain inflammatory responses without being sufficient on its own to cause disease.

Equally compelling evidence exists to support mitochondrial dysfunction as a consequence of established inflammation. In the inflamed intestine, pro-inflammatory cytokines directly impinge on the bioenergetic machinery of both the epithelial and immune cells. TNFα suppresses mitochondrial respiration in colonic epithelial cells (30), whereas IFNγ and TNFα jointly reduce the activity of respiratory complexes I and IV and impair ATP generation (31). These cytokine-mediated insults are not transient. Damaged mitochondria release ROS and mtDNA, which activate NLRP3 inflammasomes and perpetuate cytokine production, locking the system into a reinforcing circuit that persists long after the initial trigger subsides. This vicious cycle is not confined to experimental models. Analysis of the plasma from patients with IBD showed reduced respiratory complex activity and TCA cycle intermediates, which indicate mitochondrial bioenergetic failure (32) and confirm the operation of inflammation-driven mitochondrial damage in human disease.

Collectively, these observations support a model in which mitochondrial dysfunction operates at multiple levels: as a trigger in genetically susceptible individuals; as a vulnerability amplified by environmental factors; and as a consequence of cytokine-mediated damage. These roles are not mutually exclusive and probably coexist and reinforce one another over the course of the disease (**Figure 1**).

**Figure 1:**
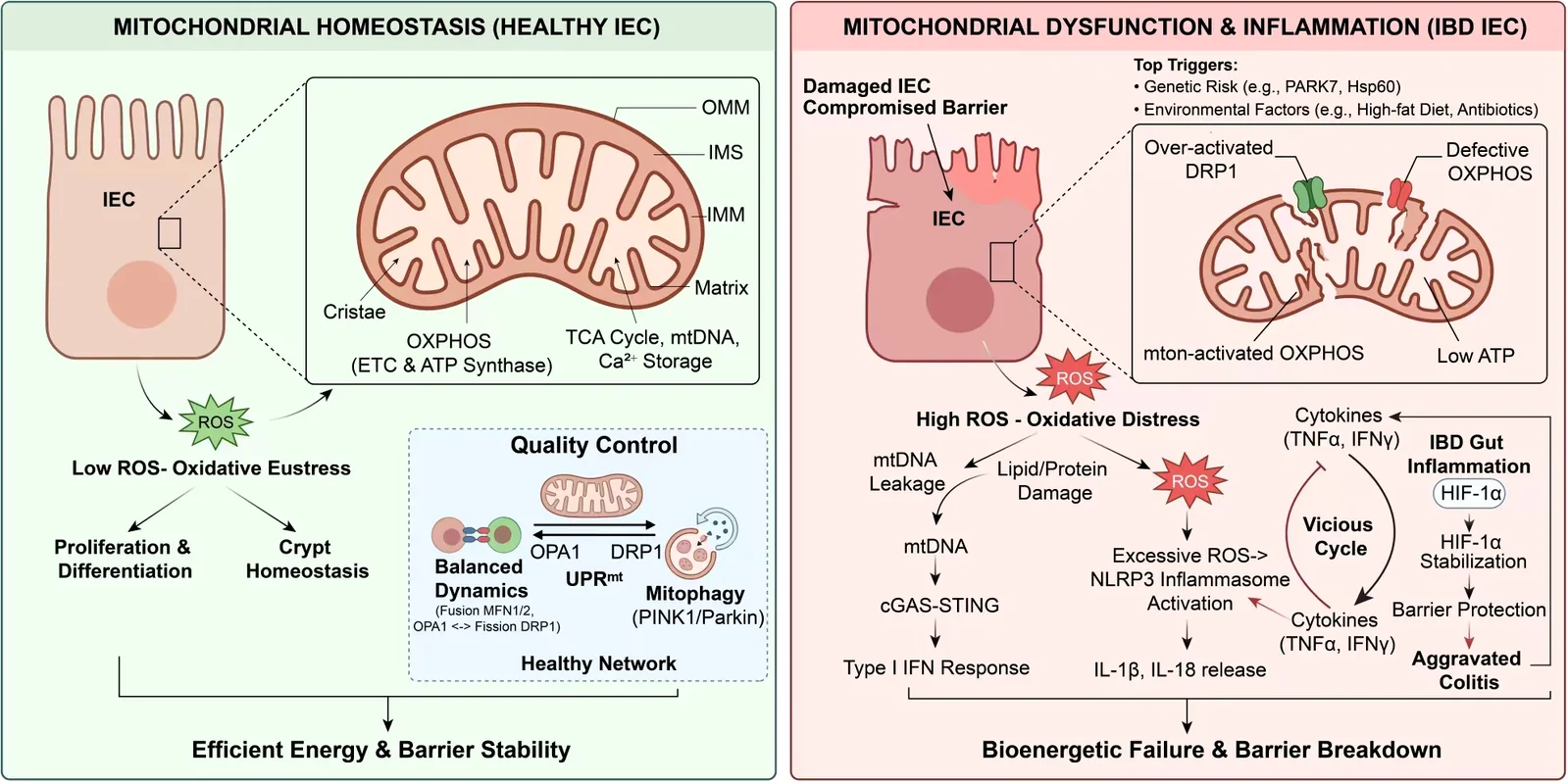
Mitochondrial homeostasis and dysfunction in IECs in IBD. The left panel illustrates mitochondrial homeostasis in healthy intestinal epithelial cells (IECs). Mitochondria maintain a dynamic network through balanced fusion (MFN1/2, OPA1) and fission (DRP1), which supports cristae integrity, efficient oxidative phosphorylation (OXPHOS), and ATP production. Physiological ROS act as signaling molecules (“oxidative eustress”) that promote IEC proliferation, differentiation, and crypt homeostasis, while PINK1/Parkin-mediated mitophagy ensures removal of damaged mitochondria. These processes collectively sustain epithelial barrier stability. The right panel depicts mitochondrial dysfunction in IBD. Genetic risk factors (e.g., PARK7, HSP60) and environmental insults (e.g., high-fat diet, antibiotics) converge to impair mitochondrial function. Excessive DRP1-driven fission disrupts the fusion-fission balance, leading to mitochondrial fragmentation and defective OXPHOS. This drives excessive ROS production (“oxidative distress”), causing oxidative damage to lipids, proteins, and mtDNA. Damaged mitochondria release mtDNA into the cytosol, engaging the cGAS-STING pathway to drive type I interferon responses, while ROS trigger NLRP3 inflammasome activation and IL-1β/IL-18 release. These inflammatory signals are amplified by pro-inflammatory cytokines (TNFα, IFNγ) from activated immune cells, which further suppress mitochondrial respiration and perpetuate epithelial dysfunction. HIF-1α stabilization in the inflamed gut exerts protective effects by upregulating barrier genes and promoting epithelial survival.

## 3 Mitochondrial glucose metabolism

### 3.1 Distinct glucose signatures in CD and UC

Although IBD is often discussed as a single entity, CD and UC show distinct metabolic profiles. In fact, these differences are apparent at the level of glucose metabolism.

Serum metabolomic studies have consistently shown that CD and UC may be distinguished from each other and from healthy controls based on their metabolic fingerprints. Metabolic alterations are more pronounced in lipid and amino acid metabolism in CD than in UC (33).

The two diseases also differ at the tissue level. UC is characterized by a more diffuse continuous inflammation of the colonic mucosa, whereas CD is transmural and affects any part of the gastrointestinal tract. Furthermore, metabolic patterns reflect these anatomical and histological differences, although active UC and CD mucosa both show increased α-glucose levels compared with that in normal mucosa, with reduced protein and carbohydrate metabolism, indicating decreased energy status and deteriorated mucosal integrity (34).

These distinctions hold direct clinical implications. For example, a metabolic intervention that works for UC may not be equally effective against CD and vice versa. Hence, precision metabolic approaches will need to account for subtype-specific differences, and this challenge is only beginning to be addressed in current therapeutic development.

### 3.2 Metabolic plasticity: beyond the glycolysis–OXPHOS dichotomy

The binary view that glycolysis is pro-inflammatory whereas OXPHOS is anti-inflammatory has long shaped the field of immunometabolism because it provides a clean, testable framework. However, the data have not fully validated this hypothesis. For example, regulatory T cells (Tregs) rely on a flexible metabolic program that includes glycolysis, OXPHOS, and fatty acid oxidation (FAO) depending on tissue context (35). Similarly, macrophages exhibit metabolic plasticity, as evidenced by the observation that M1 cells do not passively shut down mitochondrial respiration but actively rewire it (36).

These examples highlight a crucial finding that glycolysis and OXPHOS are not binary proxies for inflammatory versus anti-inflammatory function. A Treg engaged in glycolysis is not necessarily defective, and an M1 macrophage relying on OXPHOS is not necessarily anti-inflammatory.

This distinction is especially consequential in IBD. The intestinal microenvironment with its fluctuating oxygen tension, nutrient availability, and microbial metabolites demands metabolic flexibility from both epithelial and immune cells (5, 37). Therefore, in the following sections, instead of examining which cellular pathways are being utilized, we discuss the reprogramming of mitochondrial glucose metabolism in specific intestinal cell types and its contribution to IBD pathogenesis.

### 3.3 Glucose metabolism in intestinal epithelial cell subtypes

The intestinal epithelium is not a uniform metabolic landscape. It comprises at least six major differentiated cell types in addition to the intestinal stem cells (ISCs) that continuously regenerate them. Each of these subtypes exhibits a distinct metabolic program that is shaped by its position along the crypt–villus axis, its specific function, and the local microenvironment. Along this axis, a spatial metabolic gradient exists where the crypt depends less on OXPHOS and OXPHOS increases gradually as cells differentiate and move up the crypt-villus axis (38, 39). Understanding these differences directly affects the interpretation of metabolic dysfunction in IBD because inflammation does not affect all epithelial cells equally.

The evidence for these metabolic distinctions is obtained from multiple lines of inquiry. Human biopsy-based transcriptomics and single-cell RNA sequencing have begun to map metabolic heterogeneity across cell types. For example, a large transcriptomic study of pre-treatment rectal biopsies from 206 pediatric UC patients showed pronounced suppression of mitochondrial genes and function in active disease, with severity correlating with enrichment of innate immune genes (40). Mouse models with cell-type-specific deletions have provided mechanistic insight, although their relevance to human disease requires careful interpretation. Furthermore, cell lines offer experimental tractability but often fail to recapitulate the complexity of the primary tissue. Hence, the following discussion prioritizes human-derived evidence where possible and explicitly notes the model system used.

#### 3.3.1 Intestinal stem cells

ISCs occupy the crypt base and sustain rapid epithelial turnover. In the small intestine, the Paneth cells that intercalate between ISCs are highly glycolytic and provide lactate to fuel OXPHOS in adjacent stem cells, thereby establishing a metabolic partnership essential for organoid formation (41). Disruption of this metabolic crosstalk by inhibiting either the mitochondrial activity in ISCs or glycolysis in Paneth cells impairs stem cell function (41, 42). In IBD, this metabolic partnership is disrupted because mitochondrial impairment drives ISCs toward a dysfunctional Paneth cell state, a feature that predicts CD recurrence (43).

#### 3.3.2 Paneth cells

Paneth cell dysfunction in IBD is intimately linked to mitochondrial impairment. Mitochondrial dysfunction drives the transition of ISCs into dysfunctional Paneth cells, which is a feature that predicts CD recurrence (43).

#### 3.3.3 Enterocytes (absorptive cells)

As cells exit the crypt and differentiate into enterocytes, their metabolic program shifts. During intestinal inflammation, enterocytes undergo a metabolic shift toward enhanced glycolysis, as indicated by single-cell RNA sequencing data showing increased expression of glycolytic genes (*HK2, DDIT4*) in colonic epithelial cells from inflamed CD tissues (44). This shift is frequently associated with suppression of oxidative phosphorylation in the inflamed epithelium, consistent with independent evidence of impaired mitochondrial respiratory chain activity in IBD patients (32).

#### 3.3.4 Goblet cells

Goblet cell differentiation follows a similar pattern. The mitochondrial protein p32 is essential for maintaining OXPHOS and enabling goblet cell differentiation. During active inflammation, caspase-1 cleaves p32, which triggers a metabolic switch from OXPHOS to glycolysis that abrogates goblet cell differentiation (45). This mechanism links inflammation directly to barrier dysfunction in UC.

### 3.4 Metabolic features of immune cell subsets

The metabolic reprogramming observed in IBD is not confined to the epithelium. Immune cells infiltrating the inflamed mucosa undergo shifts in glucose metabolism that are tightly coupled to their functional states, and these shifts differ substantially across subsets.

#### 3.4.1 T cells (Th17 and Treg)

T cells provide the clearest example of these metabolic shifts. The Th17/Treg balance is central to IBD pathogenesis and is dependent on metabolic regulation. Th17 cells drive intestinal inflammation and are highly glycolytic. Their differentiation and effector function depend on glycolysis and the mTOR/HIF-1α axis, which together promote RORγt and IL-17 expression (46). In IBD, colonic Th17 cells exhibit enhanced glycolysis and glutamine catabolism, with impaired mitochondrial respiration that sustains pro-inflammatory cytokine production (47). Furthermore, high fructose intake promotes Th1/Th17 differentiation by activating mTORC1 through a glutamine metabolism-dependent pathway, which increases ROS levels and activates TGF-β (48). In contrast, Tregs, which are the principal anti-inflammatory subset, rely on FAO and OXPHOS to maintain their suppressive function (49). This metabolic dichotomy is not fixed. Tregs show considerable metabolic flexibility, and inflammatory signals may push them toward a Th17-like glycolytic state. This shift may contribute to loss of tolerance in IBD (46). Additionally, IL-21 complicates this landscape by reducing mitochondria–endoplasmic reticulum appositions, enhancing GSK3β activity, and perturbing pyruvate entry into mitochondria via VDAC1, thereby driving a hypermetabolic state that amplifies inflammatory responses in Treg (50).

Recently, ATP citrate lyase (ACLY) has been identified as a critical regulator of mucosal T-cell immunometabolism. ACLY was substantially downregulated in CD4^+^ T cells infiltrating the inflamed gut of patients with IBD, and ACLY-deficient T cells showed an impaired capacity to induce colitis in transfer models. Mechanistically, ACLY deficiency dampens glycolytic ATP production while enriching metabolites involved in phospholipid and phosphatidylcholine biosynthesis (51).

#### 3.4.2 Macrophages (M1/M2)

Macrophages exhibit analogous metabolic polarity. In the dynamic environment of the intestinal tract, the functions of macrophages are influenced by their metabolic adaptability to the surrounding environment, and this metabolic adaptability plays a crucial role in the pathophysiological mechanism underlying IBD (52). IL-10 is an anti-inflammatory signal that promotes a shift toward OXPHOS in intestinal macrophages through the mTORC1 pathway. The disruption of this signaling pathway damages mitochondrial glycolytic metabolism, which leads to excessive glycolysis. Subsequently, NLRP3 inflammasome activation triggers colitis (53). In patients with UC, Rubicon interacts with p22phox in the outer mitochondrial membrane. LPS activation increases this binding, leading to an increase in mitochondrial ROS production, impairment of mitochondrial complex III and biogenesis, and shifting of macrophages toward glycolysis. This metabolic switch sustains pro-inflammatory activation (54). M2 macrophages are associated with tissue repair and resolution, and they rely on FAO and OXPHOS (55). However, recent findings complicate this binary view by showing that a subset of anti-inflammatory macrophages activated by Fc receptor signaling promoted glycolysis. This challenges the conventional association of glycolysis with pro-inflammatory function alone (56).

#### 3.4.3 Innate lymphoid cells

Group 3 innate lymphoid cells (ILC3s) undergo metabolic reprogramming in IBD, although few studies support this finding. Intestinal ILC3s rely on a metabolic program that integrates glycolysis with mitochondrial ROS production to fulfill their activation demands. This metabolic rewiring sustains their proliferation and effector functions via a mTORC1- and HIF-1α-dependent process (57). Furthermore, hypoxic conditions in the inflamed intestine enhance ILC3 responses through a HIF-1α-dependent mechanism, which promotes their proliferation and activation (58).

### 3.5 Metabolic coupling between epithelium and immunity

The metabolic reprogramming of epithelial and immune cells does not occur in isolation. These two compartments communicate through metabolic intermediates that act as signaling molecules. This creates bidirectional crosstalk that amplifies inflammation.

Succinate, a TCA cycle intermediate and a key metabolite produced by intestinal flora, serves as a key example of this crosstalk (59). Mitochondrial dysfunction in IECs leads to the accumulation of succinate, which is released into the extracellular space. There, succinate binds to its G-protein-coupled receptor succinate receptor 1 (SUCNR1, also known as GPR91) expressed on immune cells such as macrophages and dendritic cells and IECs. This activates pro-inflammatory nuclear factor-κB (NF-κB) and mitogen-activated protein kinase (MAPK) signaling, thereby functioning as a metabolic checkpoint that links succinate accumulation to IBD pathogenesis (59). Additionally, it establishes a direct link between epithelial mitochondrial dysfunction and immune activation because epithelial damage generates a metabolic signal that licenses immune cells to adopt a pro-inflammatory state.

Immune-to-epithelial signals are equally important. Pro-inflammatory cytokines such as TNF-α, IFN-γ, and IL-1β are released by activated immune cells, and they directly suppress mitochondrial respiration in IECs, which impairs OXPHOS and increases ROS production (30, 31). This cytokine-mediated mitochondrial damage feeds back into the epithelial barrier defect, thereby perpetuating the cycle.

Additionally, metabolites mediate this crosstalk. Lactic acid is produced by glycolytic immune cells and hypoxic epithelial cells. It acts as a signaling molecule that influences macrophage polarization and T-cell function (60). Short-chain fatty acids (SCFAs) produced by the microbiota—particularly butyrate—act as both fuel for colonic epithelial cells and signals that modulate immune cell metabolism, including ACLY suppression in T cells (51).

The concept of a “metabolic barrier” elucidates this interdependence. Epithelial mitochondrial function maintains the physical barrier; immune cell metabolism determines the inflammatory tone; and metabolites from both sides and from the microbiota coordinate the response. Failure of any of these components causes dysfunction in the others. This explains why IBD is not simply an epithelial disease or immune disease but rather a disorder of metabolic crosstalk (**Figure 2**).

**Figure 2:**
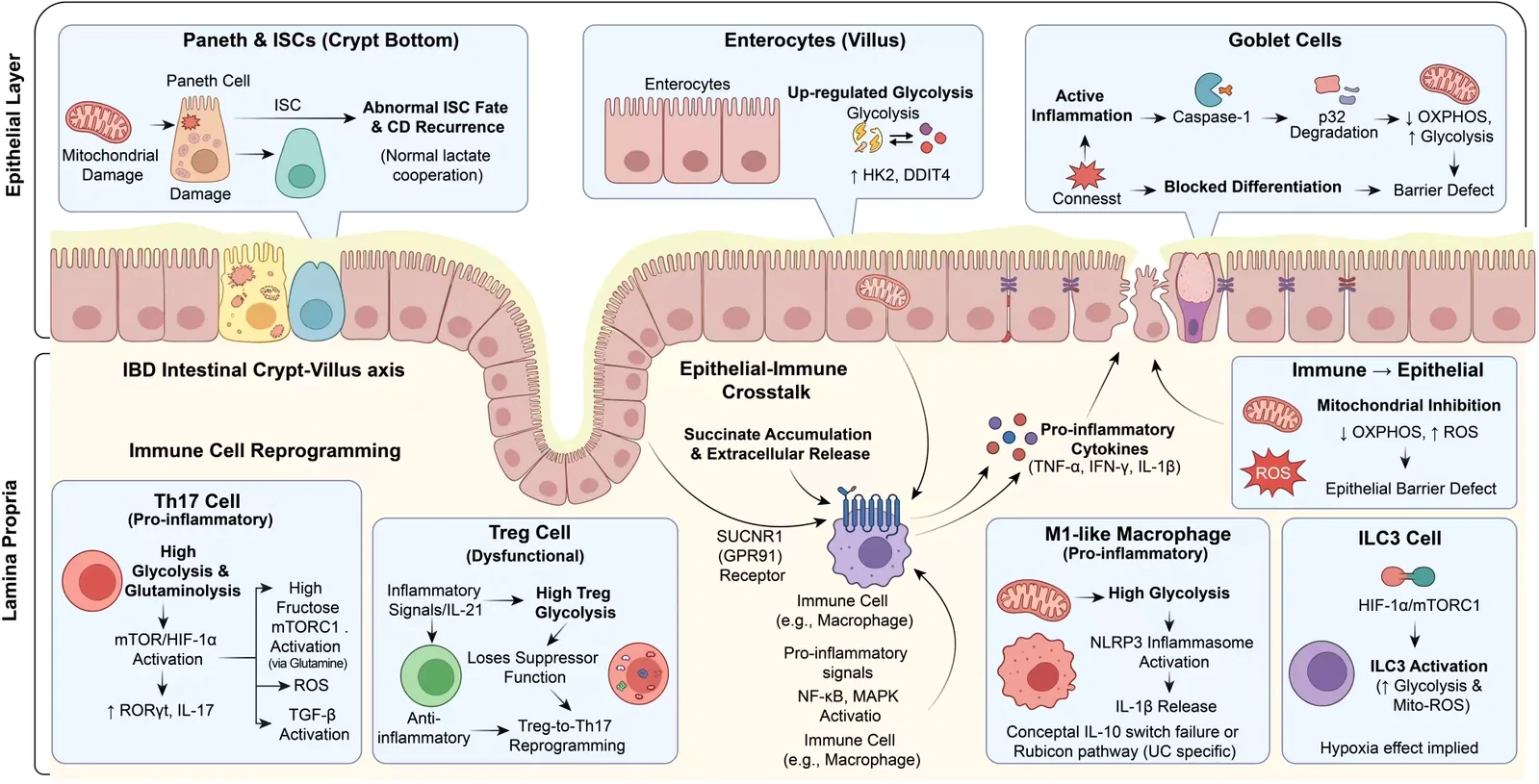
Glucose metabolism reprogramming drives epithelial and immune dysfunction in IBD. The upper panel illustrates glucose metabolic alterations across intestinal epithelial cell subtypes along the crypt-villus axis. At the crypt base, Paneth cells provide lactate to fuel oxidative phosphorylation (OXPHOS) in adjacent intestinal stem cells (ISCs); mitochondrial impairment disrupts this partnership and drives ISCs toward a dysfunctional Paneth cell state that predicts Crohn’s disease recurrence. In enterocytes, inflammation shifts metabolism from OXPHOS to glycolysis, with increased expression of glycolytic genes (HK2, DDIT4). In goblet cells, caspase-1 cleaves the mitochondrial protein p32 during active inflammation, impairing OXPHOS and blocking differentiation, which compromises mucus barrier function. The middle panel depicts metabolic reprogramming in immune cells. Pro-inflammatory Th17 cells rely on glycolysis and glutaminolysis via the mTOR/HIF-1α axis to promote RORγt and IL-17 expression. High fructose intake enhances Th17 and Th1 differentiation through mTORC1 activation and ROS-mediated TGF-β signaling. Regulatory T cells (Tregs) depend on fatty acid oxidation and OXPHOS for suppressive function, but inflammatory signals can push them toward a glycolytic Th17-like state, contributing to loss of tolerance. The lower panel shows epithelial-immune metabolic crosstalk. Mitochondrial dysfunction in IECs causes succinate accumulation and release, which binds to SUCNR1 (GPR91) on macrophages and dendritic cells to activate NF-κB and MAPK signaling. IL-10 promotes OXPHOS in intestinal macrophages via mTORC1, while its disruption drives glycolysis and NLRP3 inflammasome activation. ILC3s integrate glycolysis with mitochondrial ROS production through mTORC1- and HIF-1α-dependent mechanisms, with hypoxia further enhancing their responses.

## 4 Mitochondrial lipid metabolism

### 4.1 Distinct lipid signatures in CD and UC

In addition to their glucose metabolism profiles, CD and UC show distinct lipid metabolic profiles. Metabolomic profiling has shown that multiple metabolites involved in lipid, amino acid, and TCA cycle pathways are perturbed in IBD, with the most pronounced changes observed in CD. These findings indicate the potential contribution of disrupted lipid and amino acid metabolism to CD pathogenesis (33).

CD is characterized by more pronounced alterations in amino acid and lipoprotein metabolism than those in UC. Patients with CD exhibit a shift in LDL particle distribution toward smaller denser species. Both CD and UC show low serum concentrations of apolipoproteins A1 and A2, and these decreases correlate with elevated fecal calprotectin. Overall, the metabolic distinction between IBD subtypes is driven by alterations in amino acid and lipoprotein profiles (61).

### 4.2 Lipid metabolism in intestinal epithelial cell subtypes

FAO within mitochondria is a primary energy source for several intestinal cell types. Unlike the enterocytes in the small intestine that utilize glucose, colonocytes and goblet cells rely heavily on the mitochondrial β-oxidation of SCFAs—particularly butyrate—to generate ATP and maintain their specialized functions (62).

#### 4.2.1 Intestinal stem cells

Mitochondrial lipid metabolism is essential for ISC maintenance and regeneration. Ketogenesis is a key pathway that occurs in the mitochondria via the rate-limiting enzyme 3-hydroxy-3-methylglutaryl-CoA synthase 2 (HMGCS2) to generate beta-hydroxybutyrate (βHB) as its primary product (63). Studies on integrated single-cell and spatial transcriptomics with organoid cultures and longitudinal mucosal biopsies from patients with UC showed a pronounced loss of ISC HMGCS2-mediated ketogenesis that impeded mucosal repair. However, supplementation with βHB restored metabolic function in the UC ISCs, alleviated cellular stress, and drove epigenetic changes necessary for ISC recovery. Furthermore, reduced colonic HMGCS2 levels correlated with poor response to multiple biologic therapies in UC (63).

In addition to ketogenesis, mitochondrial FAO supports ISC function. Mitochondrial and peroxisomal fatty acid oxidation decreased in Paneth cells under conditions of oxidative stress, with consequent effects on the stem cell niche (64).

Notably, emerging evidence from patient-derived colon organoid models has revealed a more nuanced picture of epithelial metabolism in UC. Ojo et al. found that colonoids from pediatric patients with active UC show hypermetabolic features and cellular stress specifically during differentiation, driven partly by increased proton leak and excess lipid accumulation, together with heightened activation of PPAR-α and its transcriptional targets. Pharmacological inhibition of PPAR-α reduced lipid accumulation, suppressed hypermetabolism, and decreased chemokine secretion and cellular stress markers (65). These observations contrast with the conventional view that mitochondrial activity is uniformly reduced in the inflamed epithelium, and suggest that metabolic dysfunction in UC may depend on differentiation stage and cellular context. The discrepancy may arise from differences in model systems, disease stages, or differentiation stages. Together, these findings point to lipid-related metabolic dysfunction as a key feature of the pediatric UC epithelium and support the use of patient-derived colonoids for studying epithelial-targeted therapies.

#### 4.2.2 Enterocytes (absorptive cells)

In addition to metabolizing short-chain fatty acids, the intestinal epithelium is capable of oxidizing long-chain fatty acids. Acylcarnitines delivered through the intestinal lumen are taken up via apical absorption and oxidized in the mitochondria (66). In both murine colitis models and human IBD, intestinal inflammation impaired mitochondrial function in the superficial epithelium, which limited fatty acid utilization and increased the fecal acylcarnitine levels (66).

Butyrate is a short-chain fatty acid generated by commensal bacteria through dietary fiber fermentation. It is the predominant fuel for colonocytes, drives mitochondrial respiration, and supports mitochondrial biogenesis (67). Impaired mitochondrial function in enterocytes disrupts the normal processing of dietary lipids, which is a core physiological function of intestinal mitochondria (68).

#### 4.2.3 Goblet cells

Differentiation of colonic epithelial cells toward the goblet cell lineage requires a metabolic transition from glycolytic to mitochondrial oxidative metabolism. FAO is a major contributor to mitochondrial oxidation, and defects in both mitochondrial function and goblet cell differentiation have been linked to UC pathogenesis. The mitochondrial protein p32/gC1qR/HABP1 is essential for maintaining OXPHOS and enabling terminal goblet cell differentiation. Its mucosal loss creates an energy-deficient state that impairs this differentiation process in UC (45).

#### 4.2.4 Paneth cells

Paneth cell function is tightly linked to mitochondrial integrity, and disruptions in mitochondrial lipid metabolism are directly associated with Paneth cell dysfunction in CD. Among patients with CD, those with the Type I Paneth cell phenotype exhibited mitochondrial damage even in the absence of active inflammation, and this defect correlated with poor clinical prognosis (69). Ex vivo treatment of CD mucosal biopsies with the mitochondrial-targeted antioxidant Mito-Tempo restored the expression of IL-17/IL-23, lipid metabolism-related genes, and apoptotic markers to levels comparable to those of non-IBD controls. These findings support the targeting of mitochondrial damage as a viable therapeutic strategy for CD (69).

### 4.3 Lipid metabolism in immune cell subsets

Abnormal mitochondrial lipid metabolism triggers excessive and persistent inflammatory responses by providing specific lipid signals and altering the metabolic state of the immune cells. Damaged mitochondria release mtDNA, which is a powerful damage-associated molecular pattern (DAMP) that directly aggravates inflammatory responses during active IBD (70).

#### 4.3.1 T cells (Th17/Treg)

FAO is the preferred metabolic pathway for resting or memory T cells and Tregs. It is associated with the long-term survival and regulatory functions of these cells (71). IPA is a microbiota-derived tryptophan metabolite that enhances mitochondrial respiration in CD4^+^ T cells by promoting fatty acid oxidation while suppressing glycolytic flux, as observed in DSS-induced colitis models (72).

Mitochondrial fatty acid synthesis (mtFAS) and its key enzyme MECR are essential for optimal CD4^+^ T-cell activation. T cells lacking MECR exhibit impaired mitochondrial respiration, diminished TCA cycle metabolites, and intracellular iron overload, which predispose them to ferroptotic cell death. In a Th17-driven adoptive transfer colitis model, MECR-deficient T cells showed reduced pathogenicity and lower capability to induce intestinal inflammation (73).

#### 4.3.2 Macrophages (M1/M2)

Macrophage mitochondrial cholesterol metabolism is currently considered a key determinant of intestinal inflammation in IBD. Defective efferocytosis drives the accumulation of mitochondrial oxysterols, which promote ROS production and aggravates inflammatory responses in CD. Clinically, MERTK^+^ macrophage frequency correlates inversely with disease activity. At the mechanistic level, MERTK loss upregulates cholesterol synthesis in the ER and prevents degradation of the cholesterol transporter GRAMD1A, thereby facilitating ER-to-mitochondria cholesterol shuttling. Once inside the mitochondria, this cholesterol is converted into oxysterols that directly inhibit the electron transport chain and trigger ROS bursts (74). Collectively, these observations define a disease-relevant axis that connects defective efferocytosis, perturbed cholesterol homeostasis, and mitochondrial oxidative damage.

Cholesterol homeostasis and mitochondrial bioenergetics jointly regulate the activation of inflammasomes in IBD (75). In the inflamed IBD mucosa, macrophages exhibit perturbed cholesterol homeostasis, characterized by upregulation of the influx transporters LDL-R, NPC1, and StADR3 and downregulation of the efflux mediators ABCG1, SR-BI, and LXR. This imbalance in cholesterol trafficking in combination with mitochondrial dysfunction promotes NLRP3 inflammasome activation and sustains intestinal inflammation (75).

In the inflammatory environment of IBD, pro-inflammatory signals inhibit FAO and promote glycolysis. For example, TNF-α and lipopolysaccharide (LPS) downregulate CPT1a expression, which inhibits fatty acid oxidation in macrophages and promotes their polarization to the M1 phenotype (76). In fact, restoring fatty acid oxidation (for example, using CPT1a agonists) promoted the transformation of macrophages to the anti-inflammatory M2 phenotype and enhanced the function of Tregs, thereby exerting therapeutic effects in colitis models (77, 78).

Furthermore, dietary lipids directly shape macrophage function. High-fat diets impair the ability of intestinal macrophages to mount tissue-reparative responses following injury. Mechanistically, this has been linked to lipid-driven regulation of the PPARδ/BCL6 transcriptional repressor complex in macrophages, which appears to mediate the detrimental effects of dietary fat on mucosal repair and immune homeostasis (79). For example, trans-unsaturated fatty acids triggered NLRP3 inflammasome activation in macrophages and worsened experimental colitis in mice (80).

#### 4.3.3 Dendritic cells

ACSL4 is a key enzyme in long-chain fatty acid metabolism. Among the mitochondria- and ferroptosis-related genes examined in IBD datasets, *ACSL4* exhibited the strongest positive correlation with immature dendritic cell abundance, which indicates a potential link between mitochondrial fatty acid metabolism and DC function in the IBD mucosa (81).

### 4.4 Metabolic coupling between epithelium and immunity

Additionally, lipid metabolism acts as a conduit for crosstalk between the epithelial and immune cells, although this aspect has been less studied than glucose-mediated signaling. Several reports have indicated bidirectional communication through lipid-derived molecules.

Epithelial-to-immune signals: Butyrate and other SCFAs are produced by the microbiota and metabolized by colonocytes. They act directly on immune cells through GPR41, GPR43, and GPR109A (62). Butyrate suppresses ACLY expression in T cells and modulates macrophage function via GPR109A-dependent pathways (51). Similarly, cholesterol and its oxidized derivatives such as oxysterols function as signaling molecules. Mitochondrial dysfunction in epithelial cells alters cholesterol trafficking and may contribute to a lipid-rich microenvironment that shapes immune cell activation.

Immune-to-epithelial signals: The lipid metabolic state of immune cells feeds back onto the epithelium. Pro-inflammatory macrophages secrete cytokines such as TNF-α that directly suppress fatty acid metabolism in IECs (82). Mitochondrial dysfunction in immune cells amplifies ROS production, and the resulting inflammatory milieu impairs epithelial mitochondrial function. This limits fatty acid utilization and contributes to barrier dysfunction (66). mtDNA released from damaged mitochondria in immune cells acts as a DAMP that activates inflammatory pathways in the epithelium and perpetuates barrier dysfunction (70).

In this framework, epithelial FAO provides energy for barrier maintenance; immune cell lipid metabolism determines the inflammatory tone of the mucosa; and lipid-derived metabolites coordinate responses across compartments. Disruption of any of these components causes dysfunction in the others, which reinforces the assertion that IBD is fundamentally a disorder of compartmentalized metabolic crosstalk.

## 5 Mitochondrial amino acid metabolism

### 5.1 Distinct amino acid signatures in CD and UC

Serum metabolomic profiling has shown that CD and UC are distinguished not only by their lipid signatures but also by their amino acid metabolic landscapes. Targeted metabolomics has detected elevated circulating levels of tryptophan and indole-3-acetic acid (IAA) in both subtypes along with differential regulation of specific tryptophan metabolites that distinguish CD from UC (83). Serum metabolomic profiling has shown that branched-chain amino acid (BCAA) metabolism is perturbed in IBD, with more pronounced alterations in CD. Serum levels of leucine and valine differ significantly between patients with IBD and healthy participants (33, 61). These observations suggest that amino acid dysregulation is a more prominent feature of CD pathogenesis than of UC, which raises the possibility that amino acid-based biomarkers could aid in subtype discrimination and that CD may be more amenable to metabolic interventions targeting amino acid pathways.

### 5.2 Amino acid metabolism in intestinal epithelial cell subtypes

Mitochondria integrate amino acid catabolism through TCA cycle anaplerosis. Hence, the disruption of these pathways exerts profound effects on IEC function.

#### 5.2.1 Intestinal stem cells

Glutamine is the most abundant amino acid in the human body and the primary energy source and biosynthetic precursor for rapidly proliferating cells such as regenerating IECs after damage. Glutamine enters the mitochondria via membrane transporters, where glutaminase (GLS) converts it to glutamate, which generates α-ketoglutarate (α-KG) to replenish the TCA cycle. In IBD, glutamine metabolism is significantly enhanced, and increased GLS expression is observed in the intestinal mucosa of patients with active UC and CD (84). In addition to glutamine metabolism providing carbon and nitrogen sources for cell proliferation, its derivatives are involved in the synthesis of the antioxidant glutathione (GSH), which helps cells resist oxidative stress (85). Mitochondrial glutamine metabolism disorders may lead to insufficient GSH synthesis, which increases the susceptibility of these cells to inflammation-related oxidative stress (86).

HMGCS2 is a mitochondrial matrix enzyme responsible for ketone body production—predominantly βHB. Its loss in UC ISCs represents a primary mitochondrial metabolic defect that impedes mucosal healing (63). These findings establish mitochondrial ketogenesis, which is a pathway that processes ketogenic substrates including certain amino acids, as essential for intestinal epithelial function and show that it is disrupted in IBD.

#### 5.2.2 Paneth cells

Paneth cells rely on mitochondrial metabolism, with amino acid availability acting as a critical upstream determinant. The amino acid transporter SLC7A5 supports Paneth cell maturation through mTORC1 signaling. Its IEC-specific deletion reduces mTORC1 activity, depletes mature Paneth cells, and predisposes mice to experimental colitis (87). Amino acid sensing via ATF4 regulates Paneth cell antimicrobial peptide secretion, and disruption of amino acid homeostasis is linked to increased IBD susceptibility (88). In CD, mitochondrial dysfunction in ISCs drives their transition into dysfunctional Paneth cells, which implicates defective mitochondrial metabolism (including amino acid-dependent pathways) as a primary lesion in Paneth cell failure (43). This conclusion was validated by the promotion of Paneth cell differentiation by the immunosuppressant azathioprine through the enhancement of mitochondrial OXPHOS activity, which suggests that pharmacological support of mitochondrial amino acid oxidation may provide therapeutic benefit in ileal CD (89).

#### 5.2.3 Goblet cells

α-KG is a TCA cycle intermediate derived from glutamine and other amino acids. This has been shown to drive goblet cell differentiation and strengthen the colonic mucus barrier in DSS-induced colitis models (90).

### 5.3 Amino acid metabolism in immune cell subsets

Tryptophan metabolism is a critical interface between the microbiota and host immunity. Dietary tryptophan is converted by intestinal microorganisms into various bioactive indole compounds, including indole-3-propionic acid (IPA), indole-3-acetaldehyde (IAID), and IAA (91). These metabolites are crucial for maintaining intestinal barrier integrity and immune homeostasis. In IBD, dysbiosis reduces the levels of beneficial indole compounds, leading to insufficient activation of the aryl hydrocarbon receptor (AhR) and exacerbating inflammation (92).

#### 5.3.1 T cells (Th17/Treg)

The vast majority of dietary tryptophan (approximately 95%) enters the kynurenine pathway in non-liver tissues through the catalytic action of mitochondrial enzymes IDO1 or TDO2 (91). In the inflammatory environment of IBD, pro-inflammatory cytokines strongly induce IDO1 expression (93). IDO1 converts tryptophan into kynurenine, thereby reducing the local tryptophan level and inhibiting the proliferation of T cells dependent on tryptophan. Simultaneously, kynurenine and its downstream metabolites (such as quinolinic acid) exert immunomodulatory effects by activating the AhR, promoting the differentiation of Tregs, and inhibiting the activity of Th17 cells (94). Mitochondrial dysfunction may disrupt this immune regulatory circuit by affecting IDO1 activity or AhR ligand production (95).

IPA is a tryptophan-derived metabolite that acts as a “metabolic promoter” for CD4^+^ T cells, particularly regulatory T cells (Tregs). This mechanism specifically enhances mitochondrial respiration and oxidative phosphorylation in T cells through the AhR–PGC1α axis, which shifts CD4^+^ T cellular metabolism from glycolysis to OXPHOS. In an experimental colitis model, this enhanced the immunosuppressive ability of Tregs, inhibited the response of pro-inflammatory Th17 cells, and ultimately exerted a powerful protective effect (72). Additionally, IPA suppresses mTOR activity and modulates ribosome-related pathways in Th17 cells, thereby reducing IL-17A protein expression (96).

#### 5.3.2 Macrophages (M1/M2)

Serine metabolism plays a critical role in macrophage function in colitis. In both patients with IBD and in murine colitis models, serine synthesis was selectively upregulated in colonic epithelial cells. In contrast, dietary serine depletion exerted an anti-inflammatory effect in chronic colitis, and the effect has been linked to mitochondrial dysfunction in macrophages leading to impaired nucleotide synthesis and reduced proliferative capacity (97).

Arginine is converted into nitric oxide (NO) by nitric oxide synthase (NOS). The inducible nitric oxide synthase (iNOS) is highly expressed during inflammation and produces a large quantity of NO. Moderate NO levels elicit antibacterial and vascular regulatory effects; however, excessive NO reacts with ROS to form peroxynitrite (ONOO^−^), which triggers nitrosative stress and damages protein function and mitochondrial integrity. Enterocyte-derived NO protects against colitis by limiting macrophage recruitment and tissue injury, whereas immune cell-produced NO promotes macrophage activation and exacerbates inflammatory damage (98).

The deficiency of the CD98hc transporter protein hinders the development of macrophages, thereby reducing colitis severity (99). This suggests that BCAA availability influences macrophage mitochondrial metabolism and function.

### 5.4 Metabolic coupling between epithelium and immunity

Additionally, amino acid metabolism mediates communication between the epithelial and immune compartments, although this crosstalk has been less studied than its glucose- or lipid-centered counterparts. Findings from various studies indicate bidirectional signaling through amino acid-derived molecules.

Epithelial-to-immune signals: The intestinal epithelium contains a variety of amino acid-sensing receptors and transporters that detect amino acid availability and dynamically reprogram host responses to regulate intestinal physiology and immunological status (100). Dietary tryptophan is converted by intestinal microorganisms into bioactive indole compounds such as IPA, which acts on immune cells through the AhR to promote regulatory T-cell differentiation and maintain immune homeostasis (91, 101). These epithelial- and microbiota-derived amino acid metabolites function as critical signals that shape the local immune environment.

Immune-to-epithelial signals: The amino acid metabolic state of immune cells feeds back onto the epithelium. Pro-inflammatory macrophages secrete cytokines such as TNF-α that directly suppress amino acid metabolism (i.e., glutamine uptake) in IECs (102). Mitochondrial dysfunction in immune cells, as observed in the context of serine depletion, impairs nucleotide biosynthesis and reduces proliferative capacity, which are essential processes for epithelial renewal and barrier maintenance (97). Enterocyte-derived NO confers protection against colitis by restraining macrophage infiltration and limiting tissue damage, whereas immune cell-generated NO drives macrophage activation and amplifies inflammatory injury (98).

Thus, amino acid metabolism adds a third dimension to the metabolic barrier concept. Epithelial amino acid catabolism fuels barrier maintenance; immune cell amino acid metabolism determines the inflammatory setpoint; and amino acid-derived metabolites such as tryptophan catabolites and NO coordinate responses across compartments. Disruption of these pathways reinforces the inflammatory cycle, which highlights the need for therapeutic strategies that consider the metabolic interdependence of epithelial and immune cells (**Figure 3**).

**Figure 3:**
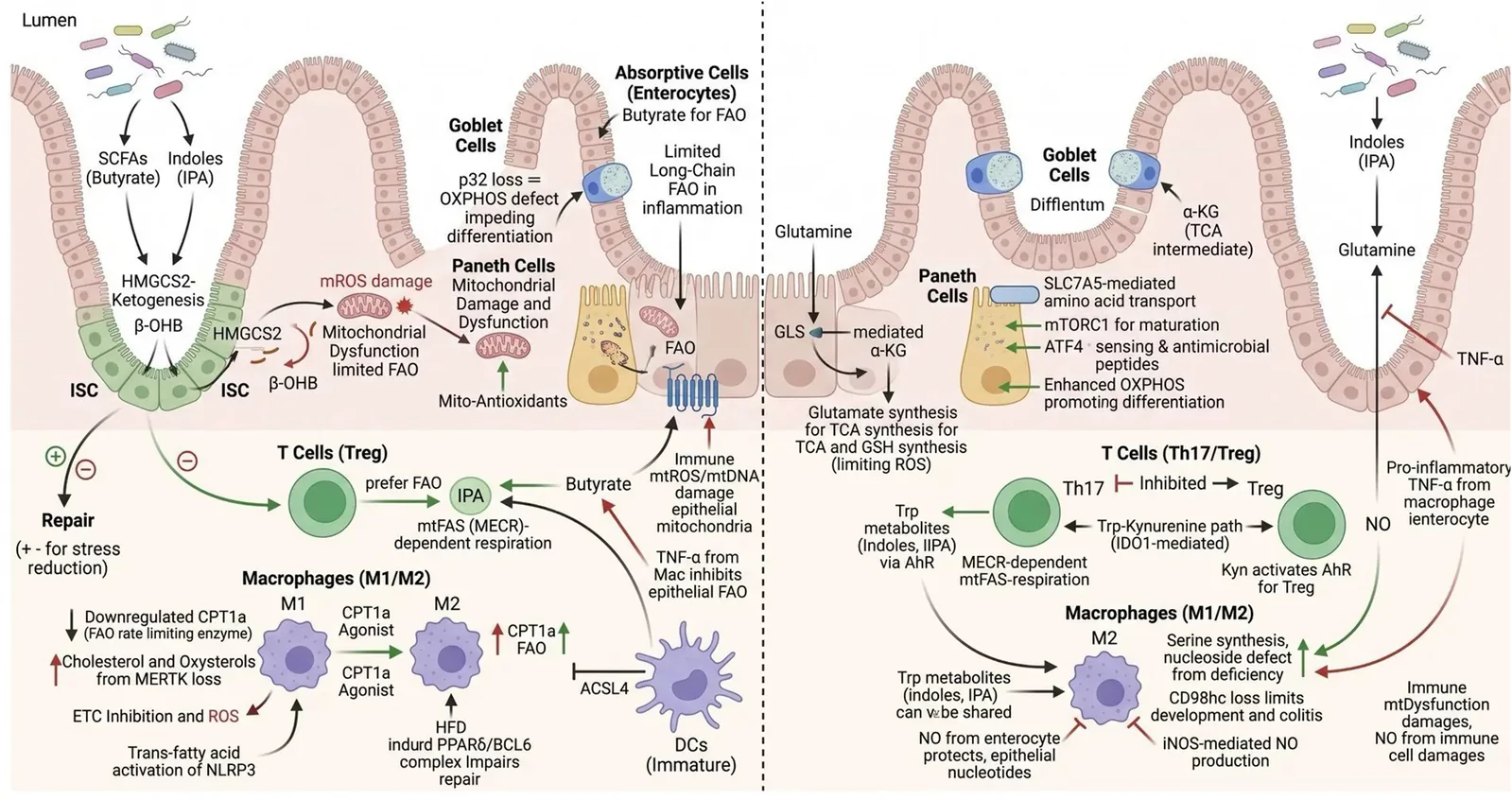
Lipid and amino acid metabolic dysregulation in intestinal epithelial and immune cells in IBD. The left panel depicts lipid metabolism. Microbiota-derived SCFAs (butyrate) fuel colonocytes via FAO. In the epithelial compartment, ISCs rely on HMGCS2-mediated ketogenesis (β-OHB) for maintenance; goblet cell differentiation requires p32-dependent OXPHOS; Paneth cells show mitochondrial damage. These epithelial alterations influence local immune responses. In the immune compartment, Tregs prefer FAO and depend on mtFAS/MECR, while M1 macrophages show downregulated CPT1a; cholesterol/oxysterols from MERTK loss trigger ROS, and trans-fatty acids activate NLRP3. The right panel depicts amino acid metabolism. Glutamine fuels the TCA cycle via glutamate to support glutathione synthesis and limit ROS; α-KG promotes goblet cell differentiation. SLC7A5-mTORC1 and ATF4 regulate Paneth cell maturation. These epithelial metabolic signals shape immune cell function. In the immune compartment, the tryptophan-IDO1-kynurenine pathway activates AhR to inhibit Th17 and promote Treg differentiation. iNOS-derived NO has context-dependent effects: epithelial NO protects, while immune NO promotes inflammation.

## 6 Integrative summary of the three metabolic pathways

The preceding sections present a detailed report of the reprogramming of glucose, lipid, and amino acid metabolism in distinct epithelial and immune cell subsets. Nevertheless, a consistent pattern may be observed across all three metabolic compartments: mitochondrial dysfunction and metabolic remodeling do not occur in isolation within a single compartment. They propagate between epithelial and immune cells through metabolic intermediates, creating a self-amplifying circuit that sustains chronic inflammation.

### 6.1 Common principles of metabolic crosstalk

Three principles recur across glucose, lipid, and amino acid metabolism. First, cell type-specific metabolic programs define the manner in which each cell subset responds to inflammatory cues. ISCs rely on mitochondrial OXPHOS and ketogenesis; enterocytes switch from OXPHOS to glycolysis during inflammation; goblet cells require p32-mediated OXPHOS for differentiation; and Paneth cells depend on amino acid transporters and mitochondrial integrity. Similarly, Th17 cells are glycolytic, Tregs depend on FAO, M1 macrophages favor glycolysis, and M2 macrophages rely on OXPHOS and FAO. Second, metabolic flexibility, which is the ability to switch between fuel sources, is not a universal property but a subset-specific trait that is most notable in Tregs and macrophages. Third, bidirectional signaling through metabolic intermediates links epithelial and immune compartments. Epithelial-derived metabolites (such as succinate, lactate, SCFAs, oxysterols, tryptophan metabolites) activate immune receptors, whereas immune-derived mediators (such as cytokines, NO, ROS) feedback to suppress epithelial mitochondrial function.

### 6.2 Glucose metabolism: the central axis of inflammatory signaling

Glucose metabolism functions as the primary axis of inflammatory metabolic crosstalk. Epithelial mitochondrial dysfunction drives a shift toward glycolysis, which reduces ATP production and compromises barrier integrity through impaired tight junctions and goblet cell differentiation. The resulting barrier leak allows microbial products to activate immune cells, which undergo glycolytic reprogramming to support Th17 and M1 pro-inflammatory phenotypes. This epithelial–immune cycle is reinforced by succinate, which is released from dysfunctional epithelial mitochondria and activates SUCNR1 on immune cells to amplify NF-κB signaling. In contrast, immune-derived cytokines suppress epithelial OXPHOS, which completes the feed-forward loop. The concept of a “metabolic barrier” captures this interdependence: epithelial glucose metabolism maintains the physical barrier; immune cell glucose metabolism determines the inflammatory tone; and glycolytic intermediates coordinate responses across compartments.

### 6.3 Lipid metabolism: fueling long-term immune function and epithelial maintenance

Lipid metabolism adds a dimension of sustained energy supply and signaling. FAO provides long-term fuel for Tregs, M2 macrophages, and colonocytes, thereby supporting immune tolerance and epithelial homeostasis. In IBD, FAO is suppressed in favor of glycolysis in pro-inflammatory subsets, whereas epithelial FAO is impaired by inflammation-driven mitochondrial dysfunction. Ketogenesis is a specialized mitochondrial lipid pathway that is essential for ISC maintenance. Its loss in UC impedes mucosal healing. Lipid-derived signals such as cholesterol metabolites, oxysterols, and SCFAs mediate bidirectional crosstalk. For example, butyrate from microbial fermentation fuels colonocytes and suppresses ACLY in T cells, whereas immune-derived inflammatory cytokines suppress epithelial FAO. The macrophage MERTK–oxysterol–mtROS axis exemplifies immune-to-epithelial lipid signaling, where defective efferocytosis drives mitochondrial cholesterol accumulation and inflammation.

### 6.4 Amino acid metabolism: integrating nutrient sensing and immune regulation

Amino acid metabolism integrates nutrient availability with immune and epithelial function. Glutamine fuels the TCA cycle in both compartments, thereby supporting epithelial proliferation and glutathione synthesis for antioxidant defense. Tryptophan metabolism is mediated by the microbiota and generates AhR ligands that promote Treg differentiation and suppress Th17 responses. In immune cells, serine supports macrophage proliferation and nucleotide synthesis, whereas arginine-derived NO exerts opposing effects depending on cellular source. That is, epithelial NO protects, whereas immune-derived NO promotes inflammation. Amino acid transporters such as SLC7A5 and ATF4-dependent sensing link amino acid availability to Paneth cell function and antimicrobial peptide secretion, thereby connecting nutrient status to innate immune defense. The immunosuppressant azathioprine enhances mitochondrial OXPHOS in Paneth cells, which suggests that pharmacological support of amino acid-dependent mitochondrial pathways may restore epithelial function in IBD.

### 6.5 Convergence and clinical implications

These three metabolic pathways converge on shared mitochondrial resources and signaling nodes. OXPHOS, TCA cycle intermediates, and ROS production are common to all three pathways. Therefore, dysfunction in any one pathway may propagate to the others through mitochondrial crosstalk. For example, impaired glucose metabolism reduces NADH and FADH_2_ production, which limits FAO and amino acid oxidation. This disrupts lipid metabolism and affects mitochondrial membrane integrity and compromises OXPHOS efficiency. Additionally, amino acid depletion impairs glutathione synthesis, which exacerbates ROS damage.

The therapeutic implications are twofold. First, cell type-specific targeting is essential because an intervention that restores OXPHOS in epithelial cells might inadvertently promote glycolysis in immune cells and vice versa. Second, the shared mitochondrial hub encompassing OXPHOS, TCA cycle anaplerosis, and redox balance suggests that strategies targeting mitochondrial function may elicit broad pleiotropic benefits across compartments, although the cell type-specific consequences of such interventions require careful evaluation of metabolic pathways. Therefore, instead of considering individual pathways in isolation, future therapeutic development must consider the integrated metabolic network that connects epithelial and immune cell function in IBD (**Figure 4**).

**Figure 4:**
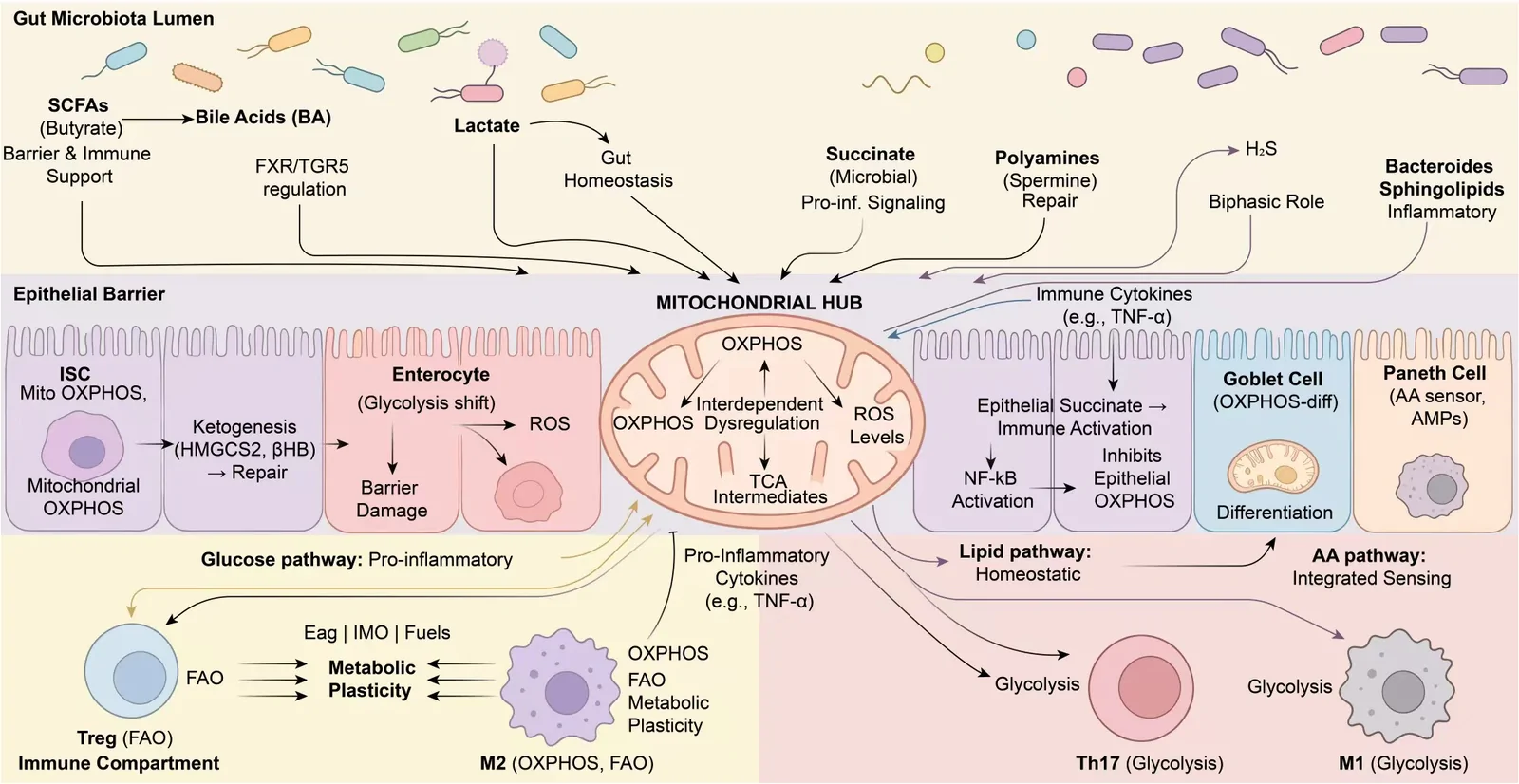
Integrated framework of epithelial-immune metabolic crosstalk in IBD. The top panel lists key microbiota-derived metabolites. SCFAs (butyrate) and bile acids support barrier function and immune regulation through FXR/TGR5. Lactate and succinate act as signaling molecules; polyamines (spermine) promote repair; H_2_S exerts biphasic effects; and Bacteroides sphingolipids modulate mucosal immunity. The center panel depicts the mitochondrial hub in intestinal epithelial cells. ISCs rely on OXPHOS and ketogenesis (HMGCS2/βHB) for maintenance; enterocytes shift to glycolysis during inflammation; goblet cell differentiation requires OXPHOS; Paneth cell dysfunction is driven by mitochondrial impairment. ROS and TCA intermediates (e.g., succinate) activate NF-κB and pro-inflammatory pathways when dysregulated. The lower panel illustrates immune cell metabolism. Th17 cells are glycolytic, Tregs rely on FAO, M1 macrophages favor glycolysis, and M2 macrophages depend on OXPHOS and FAO, with metabolic plasticity allowing contextual shifts. Arrows indicate bidirectional crosstalk: epithelial-derived signals (succinate, lactate) activate immune cells, while immune-derived cytokines (TNF-α) feed back to impair epithelial mitochondrial function, perpetuating the inflammatory cycle.

## 7 Microbiota-derived metabolite network

Glucose, lipid, and amino acid metabolism are not solely regulated by host cells. The gut microbiota continuously broadcasts metabolic signals to the host through a vast array of metabolites that extend well beyond short-chain fatty acids and tryptophan derivatives. These metabolites act as signaling molecules that modulate both epithelial and immune cell function through specific host receptors, which adds a microbial dimension to the metabolic networks discussed earlier.

SCFAs such as acetate, propionate, and butyrate exert their effects partly through metabolite-sensing GPCRs including FFAR2 (GPR43), FFAR3 (GPR41), and GPR109A (HCAR2), all of which are found on gut epithelial and immune cells (103). Additionally, butyrate functions as a signaling molecule that modulates T-cell metabolism—notably by downregulating ACLY expression (51). Butyrate supplementation enhances mitochondrial respiration and promotes epithelial barrier integrity in inflamed gut (104).

Bile acids play a dual role in immune-metabolic regulation by acting as both signaling molecules and modulators of the gut–liver crosstalk. They activate key receptors. The anti-inflammatory actions of Farnesoid X Receptor (FXR) are mediated through the suppression of NF-κB and cytokine production, whereas TGR5 signaling predominantly modulates NLRP3 inflammasome activity. Dysregulated bile acid signaling, characterized by decreased secondary bile acids in the colon and reduced FXR and TGR5 activation, leads to enhanced NF-κB transcription and promotes intestinal inflammation (105).

Succinate is a TCA cycle intermediate that accumulates upon the impairment of mitochondrial function, followed by its release into the extracellular space. Once released, succinate engages with SUCNR1 (GPR91), which is a receptor present on macrophages, dendritic cells, and IECs. This triggers downstream NF-κB and MAPK activation. This signaling cascade validates succinate as a key metabolic checkpoint in IBD pathophysiology (59). SUCNR1 recognizes succinate as an immunogenic danger cue, and its signaling has been implicated in UC pathogenesis (106). Microbial metabolism of succinate drives colonic inflammation via the SUCNR1-dependent enhancement of glycolytic flux and NF-κB activation in experimental colitis (107).

Lactate is produced by glycolytic immune cells and hypoxic epithelial cells, and it signals through G-protein-coupled receptor 81 (GPR81). Lactate-induced GPR81 activation limits NF-κB-driven MMP9 expression in IECs, thereby preserving tight-junction integrity and barrier function (108). Gut microbiota-derived lactate engages GPR81 on Paneth cells and stromal cells, which stimulates Wnt secretion to sustain the ISC niche (109).

Polyamines are aliphatic amines produced by both eukaryotic cells and gut bacteria and include putrescine, spermidine, and spermine (110). Spermidine is the most effective anti-inflammatory polyamine within the putrescine-to-spermine metabolic cascade (111). Oral spermidine attenuates colitis by inducing reparative macrophage populations and protecting the mucosal barrier (112).

Hydrogen sulfide (H_2_S) is an endogenous gaseous signaling molecule with potent immunomodulatory and cytoprotective functions (113). Although elevated H_2_S levels in the distal colon have been implicated in UC pathogenesis (114), H_2_S protected intestinal barrier integrity in experimental colitis through sulfhydration of the ribosomal protein RPS20 (115).

Sphingolipids derived from gut commensals chiefly from the Bacteroidetes phylum function both as bacterial virulence determinants and modulators of mucosal immunity. Thus, they influence Tregs and invariant natural killer T cells. Sphingolipids produced by *Bacteroides fragilis* promote colonic inflammation through dual suppression of epithelial IL-18 and ILC3-derived IL-22. Thus, this compromises mucosal immune balance and worsens colitis. Furthermore, the fecal sphingolipid profiles of patients with IBD differ substantially from those of healthy individuals (116).

## 8 Translational therapeutic strategies targeting mitochondrial metabolism

### 8.1 Preclinical strategies

A substantial number of metabolic interventions have shown efficacy in murine colitis models, although most remain at the preclinical stage.

#### 8.1.1 Targeting glucose metabolism

Among the preclinical strategies targeting glucose metabolism, several glycolytic enzymes have been identified as promising therapeutic targets. pyruvate kinase M2 (PKM2), 6-phosphofructo-2-kinase/fructose-2,6-bisphosphatase 3 (PFKFB3), and phosphoglycerate kinase 1 (PGK1) each occupy distinct nodes in the glycolytic pathway and have been evaluated in murine colitis models.

Shikonin is a natural PKM2 inhibitor that alleviated DSS-induced colitis by suppressing M1 macrophage activation and modulating glycolytic metabolism (117). Targeting PKM2 to modulate Th17/Treg balance has been validated as a therapeutic strategy for UC (118). Natural products such as Aucklandiae radix reportedly act through PKM2 to inhibit NF-κB and NLRP3 signaling in DSS-induced UC (119). PFKFB3 inhibition using the small-molecule inhibitor 3PO reduced inflammation in DSS-treated mice, and the effects were linked to gut microbiota modulation. Similarly, PFKFB3 deficiency in macrophages reduced colonic inflammation (120). PGK1 is emerging as a therapeutic target in IBD. Purine-based PGK1 inhibitors have been developed to curb glycolytic flux and lower lactate and pro-inflammatory cytokine levels, and they have shown oral efficacy in murine colitis models (121) (**Table 1A**).

**Table 1A:** Preclinical therapeutic strategies targeting glucose metabolism.

Therapeutic target/strategy	Mechanism of action	Translational stage	Clinical relevance	Limitations/adverse effects
PKM2 (Shikonin) (117)	Inhibits PKM2 dimerization/HIF-1α axis, suppresses M1 macrophage activation and aerobic glycolysis	Preclinical (DSS-induced colitis model)	Ameliorates DSS colitis	Cell-type specificity remains a concern; PKM2 may be protective in intestinal stem cells (122)
PKM2 (Th17/Treg modulation) (118)	Modulates PKM2 activity to rebalance Th17/Treg differentiation	Preclinical (UC model)	Therapeutic potential for UC	Mechanism not fully elucidated
PKM2 (Aucklandiae radix) (119)	Targets PKM2, inhibits NF-κB and NLRP3 inflammasome pathways	Preclinical (DSS-induced UC model)	Natural product-derived therapeutic candidate	Target specificity requires further validation
PFKFB3 (3PO) (120)	Inhibits PFKFB3 to reduce glycolytic flux; modulates gut microbiota composition	Preclinical (DSS-treated mice)	Attenuates intestinal inflammation	Systemic PFKFB3 inhibition may affect endothelial function
PGK1 (purine-based inhibitors) (121)	Inhibits PGK1 to suppress glycolytic ATP production and pro-inflammatory cytokines (IL-1β, IL-6)	Preclinical (orally bioavailable in murine colitis models)	IBD therapeutic candidate	Off-target effects; limited cell-type specificity (121)

PKM2, pyruvate kinase M2; DSS, dextran sulfate sodium; UC, ulcerative colitis; IBD, inflammatory bowel disease; NF-κB, nuclear factor-kappa B; NLRP3, NOD-like receptor family pyrin domain containing 3; PFKFB3, 6-phosphofructo-2-kinase/fructose-2,6-bisphosphatase 3; PGK1, phosphoglycerate kinase 1; IL, interleukin; HIF-1α, hypoxia-inducible factor 1-alpha; Th17, T helper 17; Treg, regulatory T cell.

#### 8.1.2 Targeting lipid metabolism

Lipid metabolism provides additional entry points. ACSL4 is a ferroptosis-associated target in IBD. Its inhibition using si-ACSL4 or rosiglitazone counteracted LPS-induced ferroptosis and suppressed NF-κB-mediated inflammatory responses (123). Extracts from *Scutellaria baicalensis* exerted anti-UC effects by modulating the GPX4/ACSL4 pathway and restraining ferroptosis in colonic epithelial cells (124). Pharmacological blockade of FABP5 with FABP5-IN-1 attenuated DSS-induced colitis through multiple mechanisms such as limiting pro-inflammatory macrophage accumulation, driving M2 polarization, and modulating the Th17/Treg axis (125). GPR40 (FFAR1) agonism improved DSS colitis, and this effect was attributed to increased GLP-2 production (126) (**Table 1B**).

**Table 1B:** Preclinical therapeutic strategies targeting lipid metabolism.

Therapeutic target/strategy	Mechanism of action	Translational stage	Clinical relevance	Limitations/adverse effects
ACSL4 (si-ACSL4/rosiglitazone) (123)	Inhibits ACSL4 to suppress ferroptosis and NF-κB-mediated inflammation	Preclinical (LPS-induced models)	Therapeutic potential for IBD	Target specificity remains suboptimal
GPX4/ACSL4 axis (Scutellaria baicalensis) (124)	Modulates lipid peroxidation and GPX4/ACSL4 pathway to restrain ferroptosis in colonic epithelial cells	Preclinical (colitis models)	Therapeutic potential for UC	Active components and mechanisms require further clarification
FABP5 (FABP5-IN-1) (125)	Inhibits FABP5 to reduce inflammatory macrophage infiltration, promote M2 polarization, and modulate Th17/Treg balance	Preclinical (DSS-induced colitis model)	IBD therapeutic candidate	Early-stage development; clinical translation pending
GPR40 (FFAR1 agonism) (126)	Activates GPR40 to upregulate GLP-2 production, improving DSS colitis	Preclinical (DSS mouse model)	IBD therapeutic potential	Mechanism not fully understood

ACSL4, acyl-CoA synthetase long-chain family member 4; LPS, lipopolysaccharide; GPX4, glutathione peroxidase 4; FABP5, fatty acid-binding protein 5; GPR40, G-protein-coupled receptor 40; FFAR1, free fatty acid receptor 1; GLP-2, glucagon-like peptide 2; DSS, dextran sulfate sodium; UC, ulcerative colitis; IBD, inflammatory bowel disease; Th17, T helper 17; Treg, regulatory T cell; NF-κB, nuclear factor-kappa B.

#### 8.1.3 Targeting amino acid metabolism

Amino acid pathways have yielded several candidates. Pharmacological blockade of ASS1 with the small-molecule inhibitor C-01 attenuated experimental colitis through mTOR pathway suppression. In contrast, ASS1 overexpression or arginine administration exacerbated the disease, which indicates that the ASS1/arginine axis is a disease-promoting pathway in the colonic microenvironment (127). Dietary arginine reduced colonic injury and preserved mucosal barrier integrity in DSS-induced colitis (128). Taurine supplementation exerted protective effects in colitis including inflammation suppression, barrier integrity restoration, and Th17/Treg balance restoration (129). D-alanine protected against DSS-induced colitis by inhibiting IL-12 and IL-23 production in macrophages. Notably, circulating D-alanine levels are lower in UC patients than in healthy participants (130). D-methionine supplementation alleviated disease severity and suppressed inflammatory gene expression in murine IBD models (131) (**Table 1C**).

**Table 1C:** Preclinical therapeutic strategies targeting amino acid metabolism.

Therapeutic target/strategy	Mechanism of action	Translational stage	Clinical relevance	Limitations/adverse effects
ASS1 (C-01) (127)	Inhibits ASS1 to block arginine biosynthesis and suppress mTOR pathway, attenuating colonic inflammation	Preclinical (colitis mouse model)	Colitis therapeutic candidate	Arginine supplementation may exacerbate disease (127)
Arginine supplementation (128)	Dietary arginine reduces colonic injury and preserves mucosal barrier integrity	Preclinical (DSS-induced colitis)	IBD adjunctive therapy	Efficacy requires further validation
Taurine (129)	Suppresses inflammation, restores barrier integrity, and rebalances Th17/Treg	Preclinical (colitis models)	IBD therapeutic potential	Complex multi-target mechanism; safety profile needs evaluation
D-Alanine (130)	Inhibits macrophage IL-12 and IL-23 production; serum D-alanine levels are reduced in UC patients	Preclinical + clinical correlation (DSS model + UC serum)	UC diagnostic/therapeutic potential	Mechanism not fully understood (130)
D-Methionine (131)	Alleviates disease severity and suppresses inflammatory gene expression	Preclinical (murine IBD models)	IBD therapeutic potential	Efficacy requires further validation

ASS1, argininosuccinate synthetase 1; DSS, dextran sulfate sodium; UC, ulcerative colitis; IBD, inflammatory bowel disease; Th17, T helper 17; Treg, regulatory T cell; mTOR, mechanistic target of rapamycin; IL, interleukin.

#### 8.1.4 Other preclinical strategies

Probiotics and postbiotics continue to generate interest. *Lactobacillus helveticus* R0052 attenuated DSS colitis via gut microbiota and metabolite modulation (132). *Lacticaseibacillus paracasei* L21 and its postbiotics improved barrier function and activated the HIF1α/AhR–IL-22 axis (133). Postbiotic exopolysaccharides synergized with *Lactobacillus acidophilus* to reduce intestinal inflammation (134).

Omega-3 PUFAs have shown mixed clinical results, but specific metabolites such as 4-HDHA have shown efficacy in DSS colitis through PPARγ-dependent suppression of apoptosis and inflammation (135).

Additionally, plant-derived compounds and nanodelivery systems are under preclinical development. For example, morin hydrate acted as a PPARγ and Nrf2 agonist (136). 6-gingerol exhibited anti-colitis properties (137). Curcumin encapsulated in nanostructured lipid carriers influenced macrophage phenotype switching and suppressed TLR4/NF-κB pathway activation (138). Plant-derived extracellular vesicle-like nanoparticles (PELNs) have emerged as an oral delivery platform capable of withstanding harsh gastrointestinal conditions including low pH and digestive enzyme activity (139). PELNs from Folium Artemisiae Argyi and Coix lacryma-jobi seeds have shown efficacy in colitis models (140, 141) (**Table 1D**).

**Table 1D:** Other preclinical therapeutic strategies.

Therapeutic target/strategy	Mechanism of action	Translational stage	Clinical relevance	Limitations/adverse effects
*Lactobacillus helveticus R0052* (132)	Modulates gut microbiota and metabolites to restore metabolic homeostasis	Preclinical (DSS colitis model)	IBD therapeutic potential	Strain-specific effects; complex mechanisms
*Lacticaseibacillus paracasei L21 & postbiotics* (133)	Activates HIF1α/AhR-IL-22 axis and improves barrier function	Preclinical (UC models)	UC therapeutic potential	Strain-specific effects
Postbiotic exopolysaccharides (134)	Synergizes with *L. acidophilus* to reduce intestinal inflammation	Preclinical (colitis mouse model)	IBD therapeutic potential	Mechanisms not fully elucidated
4-HDHA (omega-3 metabolite) (135)	Activates PPARγ to suppress apoptosis and inflammation	Preclinical (DSS colitis model)	IBD therapeutic potential	Metabolic stability requires validation
Morin hydrate (136)	Acts as PPARγ and Nrf2 agonist to mitigate colon inflammation	Preclinical (colitis models)	IBD therapeutic potential	Oral bioavailability requires optimization
6-gingerol (137)	Exhibits anti-inflammatory and antioxidant activities	Preclinical (DSS-UC model)	UC therapeutic potential	Stability of active components requires evaluation
Curcumin nanodelivery (138)	Influences macrophage phenotype switching and suppresses TLR4/NF-κB pathway activation	Preclinical (colitis models)	IBD therapeutic potential	Requires nanoformulation to improve bioavailability
PELNs (139–141)	Oral delivery platform resistant to extreme pH and enzymatic degradation; harnesses plant-derived miRNAs for cross-kingdom regulation	Preclinical (colitis models)	Targeted oral therapy for IBD	Scalability and safety require validation (139)

AhR, aryl hydrocarbon receptor; IL, interleukin; PPARγ, peroxisome proliferator-activated receptor gamma; Nrf2, nuclear factor erythroid 2-related factor 2; TLR4, Toll-like receptor 4; PELNs, plant-derived extracellular vesicle-like nanoparticles; 4-HDHA, 4-hydroxydocosahexaenoic acid.

### 8.2 Emerging mitochondria-targeted approaches

Additionally, ClpP activators that reprogram T-cell metabolism have been explored. NCA029 is a ClpP activator that disrupted mitochondrial respiratory complexes, inhibited OXPHOS in CD4^+^ T cells, and rebalanced Th17/Treg in experimental colitis (142).

Engineered probiotics and nanozyme systems are at an early stage of development. Prussian blue nanozyme-modified probiotics enhanced targeted colonization and alleviated intestinal inflammation in IBD models (143). Nanozyme platforms that scavenge H_2_S and ROS have been reported to regulate macrophage polarization and inhibit NF-κB, STAT3, and PKM2 pathways (144).

Furthermore, PELNs are a delivery platform that harnesses plant-derived miRNAs for cross-kingdom regulation (145). PELNs from *Zanthoxylum bungeanum* have shown efficacy in protecting macrophages from inflammation (146) (**Table 2**).

**Table 2:** Emerging mitochondria-targeted approaches.

Therapeutic target/strategy	Mechanism of action	Translational stage	Clinical relevance	Limitations/adverse effects
ClpP (NCA029) (142)	Activates ClpP to disrupt mitochondrial respiratory complexes, inhibit OXPHOS in CD4^+^ T cells, and rebalance Th17/Treg ratio	Preclinical (experimental colitis models)	Targeting T-cell metabolic reprogramming	Clinical translation requires further validation
Prussian blue nanozyme-modified probiotics (143)	Enhances targeted colonization and alleviates intestinal inflammation through nanozyme activity	Preclinical (IBD models)	IBD therapeutic potential	Clinical translation pending
H_2_S/ROS-scavenging nanozyme platforms (144)	Scavenges H_2_S and ROS, regulates macrophage polarization, and inhibits NF-κB, STAT3, and PKM2 pathways	Preclinical (IBD models)	IBD therapeutic potential	Targeting specificity requires optimization
PELNs (Zanthoxylum bungeanum) (146)	Protects macrophages from inflammatory damage via cross-kingdom miRNA regulation	Preclinical (UC mouse model)	UC therapeutic potential	Scalability, safety, and biodistribution require validation

ClpP, caseinolytic protease P; OXPHOS, oxidative phosphorylation; Th17, T helper 17; Treg, regulatory T cell; H_2_S, hydrogen sulfide; ROS, reactive oxygen species; NF-κB, nuclear factor-kappa B; STAT3, signal transducer and activator of transcription 3; PELNs, plant-derived extracellular vesicle-like nanoparticles; IBD, inflammatory bowel disease; UC, ulcerative colitis.

### 8.3 Clinically evaluated metabolic interventions

A handful of metabolic interventions have advanced to clinical testing, and some have shown signals of efficacy in IBD.

CoQ10 lowered clinical disease activity in patients with mild-to-moderate UC, although its poor aqueous solubility restricted its oral absorption (147).

SGLT2 inhibitors were originally developed for diabetes but have shown gut-protective effects. In a nationwide cohort of patients with type 2 diabetes, SGLT2 inhibitor users showed lower incidence of IBD relative to those receiving DPP-4 inhibitors (148).

Metformin is a well-established AMPK activator that has been repurposed for UC. In a 6-month randomized double-blind trial involving 60 patients with mild-to-moderate UC, metformin was administered as an add-on to mesalamine. It reduced disease activity index and the levels of serum CRP, NO, and fecal calprotectin, whereas it increased IL-10 levels (149). The effect sizes suggest that metformin may be used as an adjunctive agent in UC.

NAD^+^ precursors have been proposed as a strategy to maintain colonic barrier function (150), although instability and loss of activity under inflammatory conditions limit their use.

Butyrate supplementation has been tested in multiple RCTs. In 36 patients with active UC, sodium butyrate (600 mg/kg with meals, 12 weeks) lowered Mayo scores, erythrocyte sedimentation rate, and anxiety/depression scores relative to that observed for the placebo (151). Microencapsulated sodium butyrate has been evaluated as an add-on therapy in mild-to-moderate UC with reported safety and efficacy (152).

Nevertheless, the limited number of clinically evaluated metabolic interventions highlights a critical gap in the field: most mechanistic insights have yet to progress beyond the preclinical stage. This scarcity does not reflect a lack of therapeutic potential; rather, it highlights the substantial translational barriers discussed in Section 8.4.1. Consequently, these barriers must be addressed before metabolic therapies can be translated to patients (**Table 3**).

**Table 3:** Clinically evaluated metabolic interventions.

Therapeutic target/strategy	Mechanism of action	Translational stage	Clinical relevance	Limitations/adverse effects
Coenzyme Q10 (CoQ10) (147)	Supports electron transport chain and mitochondrial function	Clinical (mild-to-moderate UC patients)	Reduces disease activity in UC	Poor aqueous solubility → limited oral absorption (147)
SGLT2 inhibitors (148)	Regulates M1/M2 macrophage polarization and tight junction proteins	Clinical (approved for type 2 diabetes; nationwide IBD cohort study)	Lower IBD incidence observed in diabetic patients	Non-IBD specific; long-term safety requires validation
Metformin (149)	Activates AMPK to modulate immune and epithelial metabolism	Clinical (6-month RCT in mild-to-moderate UC)	Reduces disease activity index, CRP, NO, and fecal calprotectin; increases IL-10	Not IBD-specific; long-term safety in IBD requires validation
NAD^+^ precursors (150)	Restores NAD^+^ levels to maintain colonic barrier function	Preclinical/early clinical (proposed strategy)	IBD therapeutic potential	Instability and loss of activity under inflammatory conditions (150)
Sodium butyrate (151)	Serves as gut microbiota-derived metabolite with immunomodulatory and barrier-protective effects	Clinical (12-week RCT in 36 active UC patients)	Lowers Mayo scores, ESR, and anxiety/depression scores	Optimal dosing and delivery require further investigation
Microencapsulated sodium butyrate (152)	Modulates gut microbiota and promotes epithelial barrier integrity	Clinical (multi-center RCT in mild-to-moderate UC)	Safe and effective adjunctive therapy for UC	Oral delivery and targeted release require optimization

CoQ10, coenzyme Q10; UC, ulcerative colitis; SGLT2, sodium-glucose cotransporter 2; DPP-4, dipeptidyl peptidase-4; AMPK, AMP-activated protein kinase; RCT, randomized controlled trial; CRP, C-reactive protein; NO, nitric oxide; ESR, erythrocyte sedimentation rate; IBD, inflammatory bowel disease; NAD^+^, nicotinamide adenine dinucleotide.

### 8.4 Translational bottlenecks and future perspectives

#### 8.4.1 Translational bottlenecks

Despite abundant preclinical data, few metabolic therapies have reached clinical practice. The obstacles are not trivial.

Bioavailability is a recurring issue. Several metabolic modulators—particularly lipophilic compounds such as CoQ10 and curcumin—are limited by low oral bioavailability owing to poor solubility, instability in the gastrointestinal tract, and extensive first-pass metabolism (147). In the context of active IBD, the inflamed gut itself presents additional barriers such as altered pH, increased intestinal permeability, accelerated transit time, and active microbial metabolism, all of which further reduce drug absorption and stability (139).

Off-target toxicity is equally concerning. Metabolic enzymes are expressed systemically; therefore, inhibiting them broadly risks unintended effects. For example, current PGK1 inhibitors continue to face potency and pharmacokinetic limitations (121), and CoQ10 safety in combination regimens remains under-studied (147).

Immunosuppression is a major risk. Several metabolic pathways targeted for therapeutic benefit in IBD—glycolysis in particular—are additionally essential for normal immune surveillance and anti-infective responses. Long-term inhibition of glycolysis may compromise the function of effector T cells required for pathogen clearance, which would potentially increase susceptibility to infections or impair vaccine responses. Thus, the boundary between therapeutic benefit and immunosuppressive harm remains poorly defined for most metabolic targets, and few preclinical studies have systematically evaluated infectious complications.

Cell-type specificity is perhaps the hardest challenge. PKM2 inhibition may be beneficial in macrophages but harmful in intestinal stem cells where PKM2 is protective (122). An intervention that restores OXPHOS in epithelial cells might simultaneously push immune cells toward a pro-inflammatory state. Hence, achieving opposing metabolic effects in different cell types within the same tissue remains a technical hurdle that has not been solved by current approaches.

Furthermore, murine models do not faithfully reproduce human IBD. Most preclinical data come from acute DSS colitis or transformed cell lines, and these systems do not capture the chronic relapsing nature of human disease, its genetic and environmental heterogeneity, or the distinct metabolic profiles of CD versus UC (33). Drug candidates that show efficacy in acute DSS colitis often fail in human trials, which raises questions regarding the predictive validity of these models for metabolic-targeted therapies.

These five limitations, namely, poor bioavailability, off-target toxicity, immunosuppression, lack of specificity, and poor model predictivity explain the extremely low successful bench-to-bedside translation rate of metabolic therapies. Overcoming these challenges would require improved molecular candidates, delivery systems, preclinical models, and patient selection. Until these issues are addressed, metabolic interventions should be viewed as adjuncts rather than replacements for current anti-inflammatory agents.

#### 8.4.2 Future perspectives

Metabolomic profiling is useful for distinguishing patients with IBD from healthy individuals (153) and predicting treatment response to biologics (154). The concept of stratifying patients by metabolic signature is appealing for matching glycolysis inhibitors to patients with glycolytic dominance, PPARγ agonists to those with FAO defects, and NAD^+^ precursors to those with synthesis bottlenecks. However, causal relationships are rarely established, and metabolic phenotypes shift with disease activity. Hence, translating this concept into practice will require prospective validation.

The microbiome–mitochondria axis is another avenue to be explored. Microbiota-derived metabolites shape mitochondrial bioenergetics, whereas mitochondrial dysfunction alters the luminal environment and microbial community composition (155). Microbial metabolites including short-chain fatty acids, secondary bile acids, and tryptophan catabolites have shown immunomodulatory and barrier-protective effects in preclinical models (156). Postbiotic preparations provide a safer alternative to live probiotics while retaining biological activity (67).

Nanotechnology-based delivery platforms including cascade-targeting systems, dual-targeting nanocarriers, and PELNs have shown preclinical efficacy in targeting mitochondria of intestinal macrophages (157, 158). However, critical questions regarding scalability, cost-effectiveness, and manufacturing feasibility remain, and these have received little attention. Furthermore, biodistribution, toxicity, and immunogenicity profiles in humans remain largely unknown.

This review has outlined three areas where the field is poised for progress. They are patient stratification by metabolic signatures, manipulation of the microbiome–mitochondria axis, and nanotechnology-based delivery systems. Overall, these directions represent an integrated vision of precision immunometabolism, personalized metabolic therapy, microbiome-targeted interventions, and mitochondrial-targeted drug delivery systems, each of which faces substantial hurdles before clinical translation may be achieved.

## 9 Conclusion

Substantial evidence supports the hypothesis that mitochondrial dysfunction and metabolic reprogramming are not passive factors in IBD; rather, they are active participants of disease pathogenesis. The intestinal epithelium and immune cells exist in a state of metabolic interdependence where epithelial mitochondrial failure compromises barrier integrity, which in turn licenses immune cells to adopt pro-inflammatory metabolic programs. Consequently, the cytokines that they release further impair epithelial mitochondrial function. This self-amplifying cycle driven by disrupted glucose, lipid, and amino acid metabolism provides a unifying framework for understanding the connection between two seemingly distinct pathological features such as barrier breakdown and immune dysregulation at the molecular level.

This conceptual shift holds direct therapeutic implications. Rather than targeting inflammatory mediators downstream, intervening at the mitochondrial metabolism level may fundamentally restore homeostasis. Evidently, strategies that correct epithelial energy deficits, redirect immune cell fuel preference, or modulate the metabolic signals that pass between compartments have shown promise in preclinical models. The recognition that CD and UC exhibit distinct metabolic profiles adds another layer of complexity and opportunity. It suggests that subtype-specific metabolic interventions may eventually be possible.

However, a wide gap exists between mechanistic insight and clinical application owing to several unresolved questions. First, causality remains uncertain because despite the documentation of several metabolic alterations observed in IBD, whether they drive inflammation or merely reflect it is often unclear. Second, cell-type specificity is a major hurdle because an intervention that corrects metabolism in epithelial cells might inadvertently promote inflammation in immune cells and vice versa. Third, most preclinical data are derived from acute murine models that do not fully recapitulate the chronic heterogeneous nature of human IBD. This raises concerns of translational validity.

Finally, although the mitochondria constitute a critical node in IBD pathophysiology, they are not a “magic bullet.” Obtaining a definitive solution requires rigorous human validation, biomarker-driven patient stratification, and delivery systems that distinguish between cell types. These challenges are substantial; however, recent advances have provided the tools required to address these goals. Multi-omics platforms—including single-cell metabolomics and spatial transcriptomics—for patient stratification, organoid systems for mechanistic validation, and nanomedicine platforms for cell-specific delivery are promising approaches that warrant further exploration. Precision immunometabolism, personalized metabolic therapy, microbiome-targeted interventions, and mitochondrial-targeted drug delivery systems are no longer speculative concepts but active areas of investigation that may eventually translate into tangible clinical benefit.

As of now, metabolic therapies should be viewed as adjuncts to rather than replacements for current anti-inflammatory agents and be pursued with the caution warranted for any intervention targeting fundamental cellular processes.
